# So Close to a Deal: Spatial-Distance Cues Influence Economic Decision-Making in a Social Context

**DOI:** 10.1371/journal.pone.0135968

**Published:** 2015-08-19

**Authors:** Ramzi Fatfouta, Stefan Schulreich, Dar Meshi, Hauke Heekeren

**Affiliations:** 1 Department of Education and Psychology, Freie Universität Berlin, Berlin, Germany; 2 Cluster “Languages of Emotion”, Freie Universität Berlin, Berlin, Germany; 3 Berlin School of Mind & Brain, Humboldt-Universität zu Berlin, Berlin, Germany; Max Planck Institute for Human Cognitive and Brain Sciences, GERMANY

## Abstract

Social distance (i.e., the degree of closeness to another person) affects the way humans perceive and respond to fairness during financial negotiations. Feeling close to someone enhances the acceptance of monetary offers. Here, we explored whether this effect also extends to the *spatial* domain. Specifically, using an iterated version of the Ultimatum Game in a within-subject design, we investigated whether different visual spatial distance-cues result in different rates of acceptance of otherwise identical monetary offers. Study 1 found that participants accepted significantly more offers when they were cued with spatial closeness than when they were cued with spatial distance. Study 2 replicated this effect using identical procedures but different spatial-distance cues in an independent sample. Importantly, our results could not be explained by feelings of social closeness. Our results demonstrate that mere perceptions of spatial closeness produce analogous–but independent–effects to those of social closeness.

## Introduction

Imagine yourself at a negotiation table. Someone is given a sum of money and asked to share it with you. Is it possible you would evaluate the same split of money as either fair or unfair depending on the relative placement of objects on the table? Indeed, social cognition research suggests that incidental cues (e.g., spatial relations) affect higher-order cognitive and behavioral processes outside people’s awareness [1,2]. Four experiments reported by Williams and Bargh [3], for example, demonstrate how spatial-distance cues affect individuals’ evaluations and affect-based judgments. Plotting two points on a Cartesian plane that are relatively close (vs. distant) to each other led participants to feel more attached to their family and hometown. Hence, perceptions of spatial distance exert analogous influences on perceptions of social distance [4].

People commonly use spatial relations to describe interpersonal relationships (e.g., “We’ve been close for years, but we’re beginning to drift apart”; [5], p. 50), because these concepts are mentally represented in terms of space [6,7]. Specifically, conceptual metaphor theory posits that we understand more abstract concepts (e.g., social closeness) by mapping them onto our understanding of more concrete concepts, such as spatial proximity [6–8]. In fact, spatial concepts evolve early in ontogenetic development and lay a mental foundation for later-built, more abstract concepts of psychological distance (for a review, see [9]). Research on concept formation, for example, suggests that spatial concepts are experientially grounded and, hence, among the first that preverbal infants begin to understand [10]. Attachment research (e.g., [11]) further supports the natural link between both social closeness (e.g., intimacy) and spatial closeness (e.g., seeking proximity to the caregiver). This line of thought is further corroborated by recent neuroimaging data [12], suggesting that different domains of distance (e.g., spatial, temporal, and social) are encoded by a common representation—psychological distance (e.g., [13]).

Past research has demonstrated that social closeness biases economic decision-making [14]. Being close (e.g., friends) versus distant (e.g., strangers) to someone, for example, mitigates social norm enforcement (specifically, fairness norms; [15]). Using the Ultimatum Game (UG; [16]), Campanhã and colleagues [15] found that individuals accepted more offers from a friend as compared to a stranger. In the UG, one player proposes how to split a given amount of money (e.g., $10) and the other responds. If the responder accepts, the sum is divided according to the proposal. If the responder rejects, however, neither player receives anything. A well-replicated finding is that small offers (e.g., $1 or $2) are rejected about 50% of the time. This “costly punishment” indicates that individuals are not purely driven by economic self-interest but rather have a preference for fairness [17]. In the context of closeness, however, individuals tend to refrain from punishment [15]—a strategy that helps to preserve the relationship one has with the other [18,19].

Drawing on the proposed analogy between social and spatial distance [6,7], we reasoned that spatial distance will exert a similar decision bias. That is, perceptions of spatial closeness/distance should modulate individuals’ behavior in ways that parallel interactions with close/distant others. Accordingly, we hypothesized that participants cued with spatial closeness would accept more offers than those cued with spatial distance.

## Study 1

**Fig 1:**
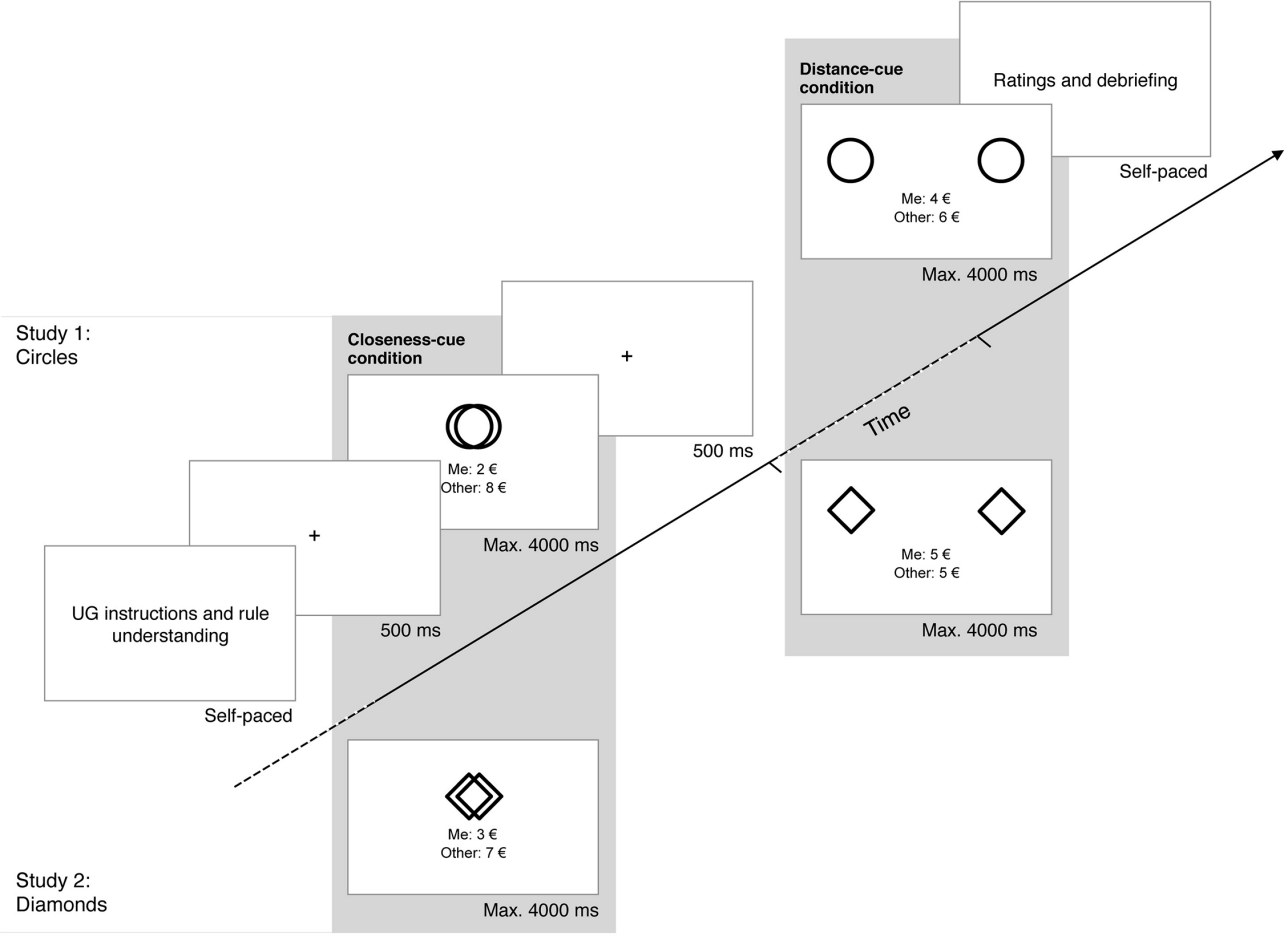
Experimental procedure and sequence of events from Study 1 and 2. The circles used in Study 1 were adapted from ([22], Fig 1, p. 597). The circles are similar but not identical to the original image, and are therefore for illustrative purposes only.

Study 1 examined the effect of spatial-distance cues on acceptance rates in the UG. We modified the cueing procedure used by Williams and Bargh [3] for two reasons: First, Williams and Bargh manipulated spatial distance by relying on both perceptual and motor representations (i.e., plotting two points on a Cartesian plane). In the present study, we manipulated spatial distance using visual cues without requiring their construction by motoric actions. Thus, any differences between conditions can be uniquely attributed to visual perceptual differences. Second, Pashler and colleagues [20] voiced concerns about the reproducibility of spatial-distance effects (but see [21]). To accommodate this, we refrained from using the original point-plotting task by Williams and Bargh [3]. Instead, we adapted visual cues that have reliably been used to represent relations among people ([22], Fig 1, p. 597]). In the present study, however, the cues made neither an explicit reference to the self or to the relation between self and other (see below).

### Method

#### Participants

The required minimum sample size was derived from power calculations (alpha = .05; power = .80) using G*Power 3.1 [23]. To detect a small effect (*f* = .10), 199 individuals are required. In addition, similar previous work using a web-based version of the UG used a sample size of around 200 [24]. To exceed this criterion and to be rather conservative, we aimed to collect around 250 valid observations.

Participants (*N* = 328) completed a web-based decision-making experiment via an online platform (SoSci Survey [25]) in exchange for a chance to win a 10€ (≈ $13.50) prize and additional payment based on their decisions in the UG. All were native German language speakers, recruited from Germany and Austria. Participants were excluded if they did not clearly understand the instructions (see below; *n* = 61; 18.60%), if they had an excessive number of non-responses (3 *SD* from the sample mean, *n* = 4; 1.22%), and if they accepted all UG offers (*n =* 20; 6.10%). Two further participants were excluded based on their comments on the study: One for having doubts that the players were real humans and one for guessing the study hypothesis. Thus, the final sample included 241 participants (*M*_age_ = 22.48, *SD =* 3.21; age range = 18–34; 73.4% females; expected power = .87). All procedures were approved by the Freie Universität Berlin institutional review board. All participants provided their written informed consent by checking a box that stated they agreed to participate in the study; participants could only then proceed with the experiment. Of note, using the full sample produced qualitatively identical results to those reported below.

#### Experimental Procedure

Participants were given detailed instructions about the UG, after which they were asked to answer four questions testing their understanding of the rules (e.g., “If you do not accept the offer, how much money do you get?”). To augment the credibility of the UG task, participants were told that they would be playing the role of the responder with two volunteers who had submitted their offers separately in a previous experiment; this procedure has been previously used in lab-based [26] and web-based [27] UG experiments. As a cover story, participants were told that each of the two proposers would be represented by a different “symbol”, to protect their identity. In fact, these “symbols” were the spatial-distance cues, consisting of two circles of the same size (3 cm) that were either overlapping (closeness cue) or non-overlapping (distance cue). To prevent any pre-game expectations associated with a particular cue, they were revealed only when the experiment began.

Participants were informed that they were playing with actual money, to elicit non-hypothetical decisions with real consequences. Specifically, they were told that one round would be randomly selected and paid out to them (random incentive mechanism). The UG consisted of 50 offers (randomized), each involving a 10€ split (see Fig 1, above the diagonal). In a within-subject design, participants received equal proportions of 5€:5€, 4€:6€, 3€:7€, 2€:8€, and 1€:9€ splits (offered: kept) from each proposer. Each trial began with a central fixation cross (500 ms), followed by the proposer’s offer. The spatial-distance cues appeared above the offer. Participants had a maximum of 4 seconds to either accept or reject the offer by pressing the *D* or *K* key on the keyboard, respectively (held constant across participants). Immediately after response selection, the next trial was presented.

Upon completion of the experiment, participants answered two questions (counterbalanced) that assessed both spatial distance (“How close are the circles in this picture?”; 1 = *not at all close*, 5 = *very close*) and social distance (“How close did you feel toward this player?”; 1 = *not at all close*, 7 = *very close*). Participants also rated the familiarity, valence, and arousal of the spatial-distance cues (counterbalanced) on a 5-point scale (1 = *not at all familiar/very negative/not at all arousing*, 5 = *very familiar/very positive/very arousing*). Finally, participants were given the opportunity to comment on the study and then fully debriefed.

### Results

#### Spatial Distance

In a check of our experimental cues, we asked participants how close the circles were for each cue. Participants perceived the circles in the closeness cue to be closer to each other (*M =* 4.50, *SE =* 0.057) than the circles in the distance cue (*M =* 1.56, *SE =* 0.056), *t*(240) = 32.940, *p <* .001, Cohen’s *d* = 3.321.

#### Economic Decision-Making

Acceptance rates (% of accepted offers) were analyzed using a 2 x 5 repeated-measure analysis of variance (ANOVA), with cue condition (closeness cue vs. distance cue) and fairness (i.e., 1€–5€) as within-subject factors. Consistent with past research on the UG [17], we found a main effect of fairness, *F*(4, 237) = 505.270, *p* < .001, η_p_^2^ = .895, with decreasing acceptance rates as offers became more unfair (*ps <* 0.001 for all post-hoc tests, Bonferroni-corrected). Importantly, there was also a main effect of cue condition, *F*(1, 240) = 4.513, *p* = 0.035, η_p_^2^ = .018. As predicted, participants accepted significantly more offers in the closeness-cue condition (*M =* 46.03%, *SE =* 0.011) than in the distance-cue condition (*M =* 45.04%, *SE =* 0.011). The fairness x cue condition interaction was not significant, *F*(4, 237) = 0.296, *p* = 0.880, η_p_^2^ = .005. Table 1 details acceptance rates across cue conditions and fairness levels.

**Table 1:** Acceptance rates in % and standard errors (in parentheses) per offer amount.

Offer	Closeness cue	Distance cue	Δ Closeness–Distance
1€	8.32% (1.39%)	6.86% (1.25%)	+1.46% (0.76%)
2€	14.77% (1.90%)	13.82% (1.82%)	+0.95% (0.92%)
3€	32.16% (2.55%)	31.91% (2.57%)	+0.25% (1.35%)
4€	78.91% (2.25%)	77.25% (2.26%)	+1.67% (1.17%)
5€	96.00% (0.94%)	95.35% (1.06%)	+0.64% (0.60%)
Average	46.03% (1.13%)	45.04% (1.11%)	+0.99% (0.47%)

Consistent with Henderson and Wakslak [28], we verified that effects still remained after accounting for other variables. It might be argued, for example, that the spatial-distance effect can be explained by the perceived social closeness. Although participants felt socially closer to the player represented by the closeness cue (*M* = 2.47, *SE* = 0.113) as compared to the player represented by the distance cue (*M* = 1.95, *SE* = 0.083), *t*(240) = 4.985, *p* < .001, Cohen’s *d* = 0.338, our results remained qualitatively identical when controlling for perceived social closeness. Again, we found a significant main effect of fairness, *F*(4, 236) = 505.716, *p* < .001, η_p_^2^ = .896, and cue condition, *F*(1, 239) = 4.539, *p* = .034, η_p_^2^ = .019, as well as a non-significant fairness x cue condition interaction, *F*(4, 236) = 0.296, *p* = .880, η_p_^2^ = .005. When controlling for familiarity, valence, and arousal, our results remained also qualitatively identical. Again, we found a significant main effect of fairness, *F*(4, 234) = 500.845, *p* < .001, η_p_^2^ = .895, and cue condition, *F*(1, 237) = 4.617, *p* = .033, η_p_^2^ = .019, as well as a non-significant fairness x cue condition interaction, *F*(4, 234) = 1.010, *p* = .403, η_p_^2^ = .017.

## Study 2

The findings of Study 1 suggest that perceptions of spatial-distance modulate economic decision-making in the UG, independent of social closeness. Given concerns about the reproducibility of spatial-distance effects (e.g., [20]), we aimed for a direct replication using an independent sample with slightly different visual cues. Again, we hypothesized that participants cued with spatial closeness would accept more offers than those cued with spatial distance.

### Method

#### Participants

The required sample size was determined as in Study 1. Participants (*N* = 296) completed a web-based decision-making experiment via the previously used online platform (SoSci Survey [25]) and using the same incentives as in Study 1. After applying identical exclusion criteria described earlier, the final sample included 273 individuals (*M*_age_ = 22.80, *SD =* 3.63; age range = 18–46; 76.6% females; expected power = .91). In this study, none of the participants reported awareness of our study hypothesis. All procedures were approved by the Freie Universität Berlin institutional review board. All participants provided their informed consent by checking a box that stated they agreed to participate in the study; participants could only then proceed with the experiment. Again, analyses based on the full sample produced qualitatively identical results to what we report here.

#### Experimental Procedure

The procedure was identical to that in Study 1, except that we used diamonds instead of circles while holding spatial distance constant across studies (see Fig 1, below the diagonal).

### Results

#### Spatial Distance

As in Study 1, participants perceived the diamonds in the closeness cue to be closer to each other (*M* = 4.55, *SE* = 0.049) than the diamonds in the distance cue (*M* = 1.65, *SE* = 0.056), *t*(272) = 33.397, *p <* .001, Cohen’s *d* = 3.344.

#### Economic Decision-Making

Again, we found a main effect of fairness, *F*(4, 269) = 497.891, *p* < .001, η_p_^2^ = .881, with decreasing acceptance rates as offers became more and more unfair (*ps <* 0.001 for all post-hoc tests, Bonferroni-corrected). The spatial-distance cues also affected participants’ choices, *F*(1, 272) = 6.575, *p* = 0.011, η_p_^2^ = .024. Consistent with Study 1, participants accepted significantly more offers in the closeness-cue condition (*M =* 43.65%, *SE* = 0.01) than in the distance-cue condition (*M =* 42.50%, *SE* = 0.01). The fairness x cue condition interaction was not significant, *F*(4, 269) = 2.301, *p* = 0.059, η_p_^2^ = .033. Table 2 details acceptance rates across cue conditions and fairness levels.

**Table 2:** Acceptance rates in % and standard errors (in parentheses) per offer amount.

Offer	Closeness cue	Distance cue	Δ Closeness–Distance
1€	7.73% (1.27%)	7.99% (1.29%)	-0.26% (0.67%)
2€	12.84% (1.65%)	11.26% (1.58%)	+1.58% (0.83%)
3€	28.75% (2.29%)	27.63% (2.24%)	+1.12% (1.22%)
4€	74.13% (2.16%)	71.06% (2.27%)	+3.07% (1.20%)
5€	94.79% (1.00%)	94.56% (1.05%)	+0.23% (0.62%)
Average	43.65% (1.03%)	42.50% (1.00%)	+1.15% (0.45%)

As in Study 1, individuals felt socially closer to the player represented by the closeness cue (*M* = 2.57, *SE* = 0.111) as compared to the player represented by the distance cue (*M* = 1.88, *SE* = 0.077), *t*(272) = 7.081, *p* < .001, Cohen’s *d* = 0.436. However, when we re-ran our analyses while controlling for perceived social closeness, our results remained unchanged. Again, we found a significant main effect of fairness, *F*(4, 268) = 498.865, *p* < .001, η_p_^2^ = .882, and cue condition, *F*(1, 271) = 6.564, *p* = .011, η_p_^2^ = .024, as well as a non-significant fairness x cue condition interaction, *F*(4, 268) = 2.334, *p* = .056, η_p_^2^ = .034. Similar holds true, when including familiarity, valence, and arousal. Again, we found a significant main effect of fairness, *F*(4, 266) = 496.630, *p* < .001, η_p_^2^ = .882, and cue condition, *F*(1, 269) = 6.603, *p* = .011, η_p_^2^ = .024, as well as a non-significant fairness x cue condition interaction, *F*(4, 266) = 2.312, *p* = .058, η_p_^2^ = .034.

## Discussion

Feeling socially close to another person modulates how humans respond to fairness during economic decision-making–specifically, by enhancing the acceptance of monetary offers [15]. Across two studies, we found consistent evidence that spatial-distance cues produce analogous effects on decision making: Compared with the distance cue, the closeness cue resulted in greater acceptance of otherwise equal monetary offers.

It is possible that participants in our studies refrained from punitive behavior because they used information about spatial closeness to simulate social closeness between themselves and the alleged proposer [29,30]. Although spatial distance has been highlighted as a mental foundation for concepts of psychological distance [4,10], results could not be explained by feelings of social closeness and persisted even after controlling for familiarity, valence, and arousal. Hence, mere perceptions of spatial closeness seem sufficient to affect economically relevant social decision-making. This dovetails with conceptual metaphor theory, which posits that more abstract concepts, in our case social closeness, are mapped onto more concrete concepts, such as spatial proximity [6–8,21,31,32]. It is also consistent with the notion that “spatial closeness is often taken to be social closeness” [33], p. 685. Changes on the concrete level (i.e., changes in spatial distance) could then operate in a similar manner as changes on an abstract level (i.e., changes in social closeness) and, hence, exert analogous influences on decision making.

Still, controlling for a single-item measure of social closeness—which may only capture part of the construct variance—does not necessarily rule out the possibility that explicit feelings of social closeness underlie the observed effects. Moreover, it might be that participants automatically accessed information about social closeness [34] that is not reflected in their explicit (i.e., deliberative) self-reports. For instance, Pöhlmann and Hannover [35] showed that explicit and implicit measures of social closeness differ in their associations with mental representations of others. Another study by Karremans and Aarts [36] showed that subliminally cueing individuals with the name of a close (vs. non-close) other person produces higher forgivability judgments. This indicates that closeness at an implicit (i.e., non-deliberative) level is associated with pro-relational responses (in our case, refraining from punishment by accepting more offers). Future studies using implicit measures and subliminal cueing might explore this possibility in greater detail.

Our results add to the limited literature on the effects of spatial-distance cues on human judgment [3], extending it in three important ways: First, by demonstrating that spatial distance also affects economically relevant choices in social decision-making and, second, by relying on a novel spatial task that does not require motoric actions. Therefore, spatial-distance effects can uniquely be attributed to visual perceptual differences. Third, we used–for the first time–a sequential (i.e., within-subject) design for studying the impact of spatial distance on people’s choice behavior. The latter point is especially crucial, as it allows generalizability to real-life interactions [37]: a human in the social world reacting to a change in spatial distance, not two (groups of) humans in separate social worlds with different spatial distances. Future studies might examine to what extent a change in spatial distance also affects decision making in one-shot UG encounters or other economic games, such as the dictator or trust game.

To note, while the effects resulting from our spatial manipulation were rather small in size, our large samples allowed us to detect and replicate the spatial-distance effects with sufficient precision [38]. Even small effects can be theoretically meaningful [39], especially when produced with minimal manipulations [40]. Practically speaking, small effects can have large economic consequences. For example, in big businesses a 1% increase in trade agreements may result in millions of dollars of additional profits. Hence, demonstrating that even a subtle visual manipulation (i.e., changing spatial-distance cues) accounts for variance in seemingly unrelated decisions adds to the theory’s practical value of spatial distance.

In conclusion, our research resonates with theories of spatial distance in which perceptual representations of physical distance affect human behavior. Moreover, our findings point to the value spatial concepts such as “close” and “distant” have for a more comprehensive understanding of social decision-making. Simply presenting identical monetary offers with different spatial cues has significant effects on judgment and decision making. That is, incidental spatial information that seems unrelated to the decision at hand can influence the very same decision.
